# Snake Bites

**Published:** 1880-08-20

**Authors:** Joseph Jones

**Affiliations:** Georgia


### Southern Medical Record.

EDITORS:

T. S. POWELL, M.D. W. T. GOLDSMITH, M.D. R. C. WORD, M.D.

*R. C. WORD, M.D., Managing Editor.*

☞All Communications and Letters on Business connected with the RECORD must be addressed to the Managing Editor.

VOL. X. ATLANTA, GA., AUGUST 20, 1880. No. 8.

### ORIGINAL AND SELECTED ARTICLES.

### SNAKE BITES.

BY JOSEPH JONES, M.D., OF GEORGIA.

The few contributions to our Journals on this subject will be a sufficient apology for offering for publication the following article. Indeed these cases seem to have been turned over, in a great measure, to empiricism. Yet there is no class of cases in which it is necessary for the attending physician to have his principles of treatment more definitely fixed and settled than in this.

A snake bite is like the shock of an earthquake in a family. The neighbors flock in, and each one who comes to witness the wonder and tell "snake stories" brings with him a "rattle snake master," an infallible remedy which he has known to cure in, at least, one case, and never known to fail, and which he urges with all the pertinacity of empirical ignorance, while the heads of the family and friends of the patient seem to be embarrassed, only in regard to which of these wonderful cures they will give preference.

Under these circumstances the physician will require more moral courage to keep control of the case than would be necessary to clear a ship's deck of a mutinous crew. And if he is not a man of firmness and well fortified with fixed principles of treatment, he will be swept off by some one of the xecessive gusts of indignation at his obstinacy in not abandoning his own remedies, supported as they are by the recorded testimony of the profession, and trying some one of these infallible cures which, at least, "could do no harm."

*Treatment.*—Immediately on arriving we give whisky, brandy or some alcoholic liquor, repeated every few minutes until the patient is completely under its influence. This will take an astonishing quantity. In a very bad case which we recently treated (the bite of a mocassin), a little girl about ten years of age drank, within a few hours, considerably over a pint of whisky with but little apparent effect.

We know this treatment is objected to by Prof. Payne (of Southern Medical College, Atlanta, than whom no higher authority exists), on the ground that it possesses no properties which can chemically act on the poison. It is a settled principle, I believe, that absorption goes on in an inverse proportion to arterial action. The whisky then prevents to a great extent, the absorption of the poison, while it sustains the sinking powers of life, which are so materially depressed in these cases, thus meeting two important indications in the treatment. We then bathe the swollen parts with volatile liniment (hartshorn and sweet oil), adding a small portion of laudanum, and envelope it in a clay poultice. This poultice, we think, acts by capillary attraction, and is the best we have ever used in such cases. After these measures have been attended to we prescribe large doses of carb. of ammonia, from thirty to sixty grs. of the powder, in half glass of sweet milk, or a fluid drachm of the spirits of ammonia in the same vehicle. The ammonia sustains the patient's strength and acts chemically in neutralizing the poison, and should be repeated, at first, as often as circumstances require, and then be continued every few hours until the poison is entirely eradicated from the system.

